# Life Course Patterns of Multimorbidity Among Black Residents in the California Health Interview Survey

**DOI:** 10.1093/geroni/igaf122.1822

**Published:** 2025-12-31

**Authors:** Xumeng Yan, Angela Gutierrez, Bo-Kyung Elizabeth Kim, Courtney Thomas Tobin

**Affiliations:** University of California, Los Angeles Fielding School of Public Health, Los Angeles, California, United States; Western University of Health Sciences, Pomona, California, United States; University of California, Los Angeles, Los Angeles, California, United States; University of California, Los Angeles Fielding School of Public Health, Los Angeles, California, United States

## Abstract

Multimorbidity, defined as having two or more chronic conditions, may exacerbate aging-related health disparities. However, the patterns of multimorbidity among Black Americans (BAs) over the life course remain less understood. Utilizing data from the 2023 California Health Interview Survey (CHIS), this study examined age- and gender-specific patterns of multimorbidity among BAs (n = 971) across six chronic conditions: diabetes, hypertension, asthma, stroke, heart disease, and high cholesterol. Logistic and Poisson regression models were used, adjusting for education level, poverty level, marital status, employment status, and health insurance status. Overall, 32.6% of BAs reported multimorbidity, and they consistently exhibited higher prevalence and mean counts of multimorbidity across all age groups compared to non-Hispanic White adults. In young adulthood (18-34 years), 11.1% of BAs reported multimorbidity, with hypertension and asthma being the most common co-occurring conditions. In middle adulthood (35-49 years), multimorbidity prevalence increased to 18.2%, with hypertension and diabetes emerging as the most frequent combination. By ages 50-64 years, 49.1% of BAs experienced multimorbidity, with hypertension and high cholesterol being the most prevalent co-occurring conditions. In older adulthood (65+ years), the multimorbidity burden remained high (45.7%), with hypertension commonly co-occurring with diabetes, high cholesterol, and asthma. Gender differences were also observed: men had higher multimorbidity rates in early and middle adulthood, while women faced a greater burden of multimorbidity later in life. These findings highlight the disproportionate burden of multimorbidity among Black women aged 50-64 years, underscoring the need for targeted preventive interventions tailored to age and gender.

